# Nebraska Veterinary Medical Association1Reported for the Journal by A. T. Peters.

**Published:** 1896-05

**Authors:** 


### NEBRASKA VETERINARY MEDICAL ASSOCIATION.[1]

THIS Association met January 13th at the Merchants' Hotel, Omaha, at 8 P.M. The members present were: Drs. A. T. Peters, Lincoln; H. L. Rammacciotti, Omaha; S. E. Cosford, Omaha; J. Anderson, Seward; V. Schaeffer, Tekamah; G. R. Young, Omaha; P. Tucker, Lincoln; J. L. Blackwell, Omaha; and A. T. Everett, South Omaha.

The first business taken up was the election of officers for the ensuing year, 1896, and the following gentlemen were appointed, viz.: Dr. A. T. Peters, President; Dr. J. Anderson, Vice-President; Dr. J. E. Blackwell, Treasurer; and Dr. A. T. Everett, Secretary. The Board of Censors was composed of the following gentlemen: Drs. H. L. Rammacciotti, G. R. Young, and V. Shaeffer.

After the election of officers Dr. A. T. Peters called the meeting to order and instructed the Secretary to read the "Constitution and By-laws" of the Association, and it was agreed that the constitution be adopted as heretofore, with the exception that the first meeting be held at the latter part of August, during the State fair week.

It was moved and seconded that a committee be appointed to draft a diploma of the Nebraska Veterinary Medical Association. The committee to consist of three members and the President, viz., Drs. J. E. Blackwell, H. L. Rammacciotti, G. R. Young, and A. T. Peters.

Other routine business having been gone through, the President then called on Dr. V. Schaeffer to read a paper on "Actinomycosis," which the Doctor handled in an able manner, and which called forth a very animated discussion on this important subject. Dr. Blackwell was then called on by the President to make a few remarks on the general outline of the "Federal Meat-inspection," which the Doctor kindly did. After a vote of thanks had been extended to the President, Dr. A. T. Peters, for the trouble and interest he had taken in the reorganization of the Association, the meeting adjourned.

[1] Reported for the JOURNAL by A. T. Peters.

